# Vitamin D and Vitamin D3 Supplementation during Photodynamic Therapy: A Review

**DOI:** 10.3390/nu14183805

**Published:** 2022-09-15

**Authors:** Anna Mazur, Katarzyna Koziorowska, Klaudia Dynarowicz, David Aebisher, Dorota Bartusik-Aebisher

**Affiliations:** 1 Students Biochemistry Science Club URCell, Medical College of the University of Rzeszów, 35-959 Rzeszów, Poland; 2 Students English Division Science Club, Medical College of the University of Rzeszów, 35-959 Rzeszów, Poland; 3 Center for Innovative Research in Medical and Natural Sciences, Medical College of the University of Rzeszów, 35-310 Rzeszów, Poland; 4 Department of Photomedicine and Physical Chemistry, Medical College of the University of Rzeszów, 35-959 Rzeszów, Poland; 5 Department of Biochemistry and General Chemistry, Medical College of the University of Rzeszów, 35-959 Rzeszów, Poland

**Keywords:** calcitriol, topical application, photodynamic therapy, cancer treatment

## Abstract

Photodynamic therapy is an unconventional yet increasingly common method of treating dermatological diseases and cancer that is implemented more often in adults than in children. Current clinical uses include treatment of actinic keratosis, superficial basal cell carcinomas, and acne. Despite its high efficiency, photodynamic therapy support supplements have recently been reported in the literature, including calcitriol (1,25-dihydroxycholecalciferol), the active form of vitamin D, and vitamin D3 cholecalciferol. In clinical trials, photodynamic therapy enhanced with vitamin D or D3 supplementation has been reported for treatment of squamous cell skin cancers, actinic keratosis, and psoriasis. Experimental research on the effect of photodynamic therapy with vitamin D or D3 has also been carried out in breast cancer cell lines and in animal models. The aim of this review is to evaluate the usefulness and effectiveness of vitamin D and D3 as supports for photodynamic therapy. For this purpose, the Pubmed and Scopus literature databases were searched. The search keyword was: “vitamin D in photodynamic therapy”. In the analyzed articles (1979–2022), the authors found experimental evidence of a positive effect of vitamin D and D3 when used in conjunction with photodynamic therapy. An average of 6–30% (in one case, up to 10 times) increased response to photodynamic therapy was reported in combination with vitamin D and D3 as compared to photodynamic therapy alone. Implementing vitamin D and D3 as a supplement to photodynamic therapy is promising and may lead to further clinical trials and new clinical methodologies.

## 1. Introduction

Photodynamic therapy (PDT) is a minimally invasive and modern therapeutic method that is clinically approved for the treatment of some oncological skin diseases, such as Basal Cell carcinoma [1,2,3], actinic keratosis [4], Bowen’s disease [5], cutaneous lymphomas [6], and cutaneous Kaposi sarcoma [7]. It has been approved for the treatment of several types of cancer, including those of the prostate [8], lung [9], breast [10], larynx [11], and bladder [12], and for the treatment of non-oncological dermatological diseases such as psoriasis [13], acne [14,15], herpes simplex labialis [16], cutaneous leishmaniasis [17], Darier disease (Keratosis follicularis) [18], and port-wine stains [19].

The main determinant of PDT is the application of a photosensitizer (PS) (which accumulates in tissue or is present in the bloodstream at the time of illumination), local illumination of a targeted site with laser light, and molecular oxygen [20,21,22,23].

Photodynamic therapy is a two-step process: 1. administration of a PS (on the surface of a lesion for skin diseases, or intravenously) [24]; 2. local illumination of the tumor or lesion with light that corresponds to the PS absorption wavelength [25]. Light is absorbed by the PS and results in the generation of reactive oxygen species (ROS) such as hydroxyl radicals or singlet oxygen (^1^O_2_), which are highly reactive and cytotoxic species [26,27,28,29].

**Figure 1:**
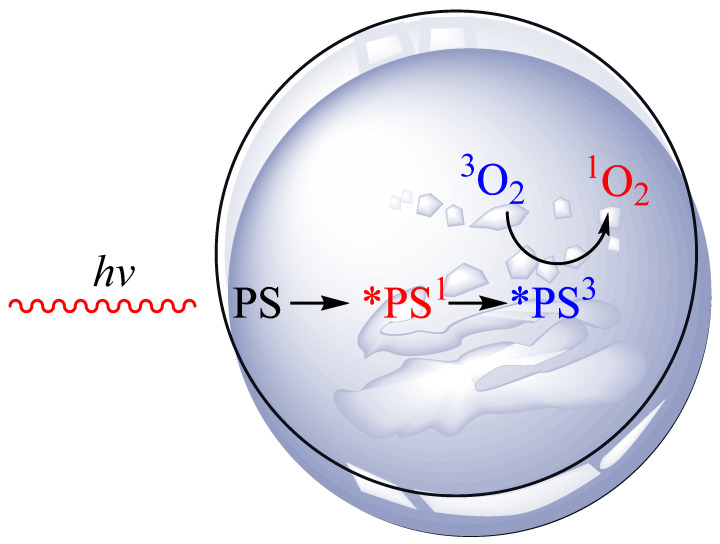
Singlet oxygen generation in vitro.

Photodynamic pathways are divided into two main types: Type I and Type II [30]. Type I involves photoinduced electron transfer, leading to local formation of superoxide, hydroperoxyl, and hydroxyl radicals. Type II is characterized by energy transfer from the PS in its excited triplet state to oxygen, producing cytotoxic ^1^O_2_ [31]. Figure 1 shows a generalized scheme for the production of singlet oxygen in vitro. The in vitro research methodology allows for a better understanding of the mechanism of action of PDT and its more effective use in in vivo research.

For the photodynamic effect to take place, three elements are necessary: an administered photosensitizer (PS), a light source (*hv*), and oxygen (O_2_). An example of an in vitro Type II process is presented above. Cancer tissue has been placed in a petri dish. A specifically selected PS with an appropriate concentration is applied. The tissue is then exposed to laser light of a given wavelength. Absorption of a laser light quantum by PS produces a short-term singlet excited state (*PS^1^). This state can lose energy through fluorescence, internal conversion to heat, or through an intersystem transition to a long-term triplet excited state (*PS^3^). The (*PS^3^) transfers energy to the triplet oxygen. As a result of this process, the triplet oxygen (^3^O_2_) is converted into singlet oxygen (^1^O_2_) via triplet–triplet energy transfer, which can induce cancer cell death.

Improvements to PDT are currently addressing the difficulty in treating areas located in deeper tissue due to the limited penetration of light [32,33]. Research is also being conducted on the modification of photosensitizers in order to minimize their impact on a specific organ and eliminate the potentially harmful effect of PDT on healthy tissue [33]. Enhanced PDT is also practiced through the use of nanoparticles bearing PS [34], chemotherapeutic agents [35], or in conjunction with calcipotriol (a synthetic derivative of calcitriol) [36].

Vitamin D3 and each of its analogues is a steroid hormone involved in the metabolism of calcium. It has been shown in experimental studies that this supplement increases the production of protoporphyrin IX in mitochondria, supporting the destruction of cancer cells during PDT [37]. It is noteworthy that the mechanisms by which vitamins D and D3 (and their analogues) may influence PDT response are not fully understood. Understanding how high doses of vitamins D and D3 affect PDT reactivity continues. The analysis of the influence of calcitriol on the physiology of the organism and cells is very often analyzed in in vitro studies. It is now known that calcitriol promotes the repair of UV-induced mutations in keratinocytes by increasing functional P53 and has several anti-tumor effects on epidermal tumors through the immune system. The transcriptional profile of calcitriol-treated healthy keratinocytes was examined, showing upregulation of approximately 82 genes and downregulation of 16 other genes [38]. One example of the analysis of the effect of calcitriol on the patient’s body is the study conducted by Anand et al. Tumors pretreated with calcitriol showed increased apoptotic cell death following PDT. Tumors pre-conditioned with calcitriol before receiving ALA-PDT showed greater activation of the external apoptotic pathway (greater caspase-8 cleavage and increased TNFα production) [39]. There are also known cases of in vitro experiments in which cells treated with calcitriol increased the degree of DNA fragmentation compared to untreated cells [40]. For example, treatment with calcitriol can reduce stemness to varying extents in a panel of glioblastoma stem-like cells, and that it effectively hinders tumor growth of responding glioblastoma stem-like cells ex vivo [41].

The aim of this review was to evaluate the reports on the usefulness and effectiveness of topical synthetic vitamin D in support of PDT in both adults and children.

## 2. Materials and Methods

### 2.1. Search Strategy and Select Criteria

A search focused on the effect of synthetic vitamin D and D3 on the effectiveness of PDT was conducted on Pubmed and Scopus from inception (1979) to June 2022. This review was conducted based on the Preferred Reporting Items for Systematic Reviews and Meta-Analyses (PRISMA) guidelines [42]. The search term included the phrase: “vitamin D in photodynamic therapy”. Two authors undertook the task of identifying relevant data. Any discrepancies between the reviewers were resolved by a third author. The authors of this review worked on the basis of an agreed scheme, selecting articles based on their title, language (English), abstract, and access. Duplicate records were removed.

Full-text and accessible articles were reviewed. In order to minimize the selection bias, the inclusion and exclusion criteria were established as follows:

Inclusion criteria:

- vitamin D supplemented or administered during PDT treatment;
- analysis of the effect of synthetic vitamin D supplementation on the effectiveness of PDT;
- clinical and experimental studies of the effect of vitamin D supplementation on the effectiveness of PDT in animal studies or in in vitro studies on cell lines;
- dermatological diseases, cancer treatment, and treatment of internal organ tumors

Exclusion criteria:

- no analysis of the relationship between vitamin D supplementation and PDT effectiveness
- conducting photochemotherapy

### 2.2. Data Extraction

Two authors identified relevant information according to the inclusion and exclusion criteria. From the qualified articles, they listed the publication year, type of disease, type of examination, and the vitamin D or D3 used as a supplement to PDT.

## 3. Results

### 3.1. Study Selection

**Figure 2:**
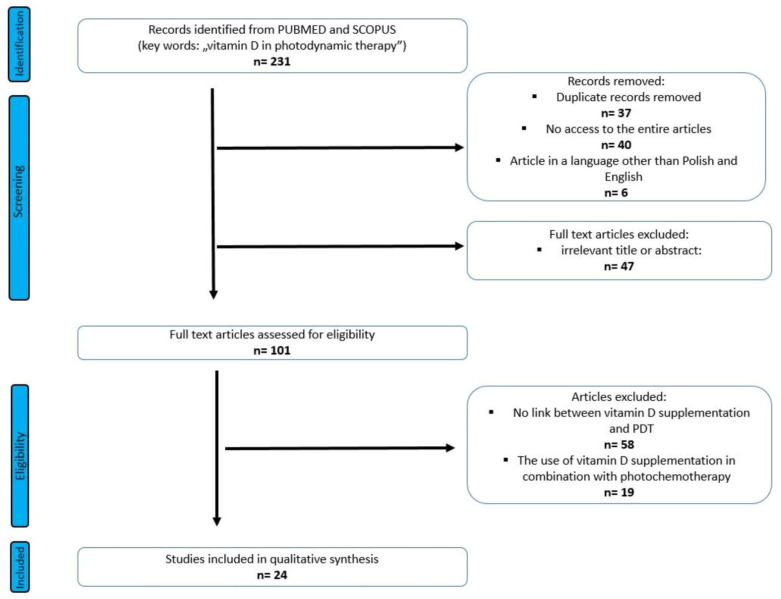
PRISMA flow diagram of included studies.

We searched 231 articles, and 24 articles were selected for this review (Figure 2). Among the included, 19 were research articles and 5 were reviews.

### 3.2. Study Characteristics

The 24 articles included in the review were analyzed in terms of the type of study (clinical study/animal study/cell lines), type of disease, type of application, and effect of synthetic vitamin D administration on PDT efficacy. An additional analyzed parameter was the type of PS used. Table 1 shows a description of study characteristics.

### 3.3. Results of Studies

Of the 20 studies analyzed, 45% were clinical human studies, 30% were in vitro studies on cell lines, and 25% were animal studies. In all clinical trials, patients were adults aged ≥18 years. The most common type of diseases treated was actinic keratoses (25%), followed by squamous cell skin cancers (15%). Cell line in vitro treatment on breast, glioma, and prostate cells represented 25%. Overall, 55% of synthetic vitamin D or D3 was administered topically (using a cream or ointment), 30% was added to cell cultures, and 15% was added systemically or intraperitoneally—in two cases, orally (as a drug or dietary). For PDT, the most frequently used PS was 5-ALA (in 60% of cases), followed by MAL (in 30%). Table 2 presents a summary of the studies reported in this review.

## 4. Discussion

Vitamin D is a fat-soluble vitamin that has a significant impact on human functioning and health [59]. A natural source of vitamin D for humans is its synthesis in the skin under the influence of ultraviolet B (UVB) radiation (290–315 nm) [60]. Previtamin D occurs mainly in the spinous and basal layers of the epidermis, with fewer resources in the outer layers of the epidermis or dermis [61,62,63].

Vitamin D therapy using calcitriol, calcipotriol, and tacalcitol has been used to treat skin conditions including psoriasis, atopic dermatitis, and vitiligo [64]. Effective treatment of these cases with synthetic vitamin D or D3 continues to be clinically relevant [65]. The possibility of an enhanced PDT treatment is provided by vitamin D and D3 supplementation. A common hypothesis is that PDT with adjuvant vitamin D therapy is more effective than PDT alone [66]. It has been suggested that vitamin D and D3 modifies the tumor environment, allowing for a reduction of effective dose of the administered PS [67]. The motivation to analyze correlations between vitamin D and D3 and the effectiveness of PDT was the reported differentiated tumor responses to PDT in the presence of vitamin D and D3 [68,69,70]. In order to confirm the hypothesis, over the last 43 years, research and review articles have been written assessing the effect of vitamin D and D3 on the effectiveness of PDT. Researchers introduced vitamin D-fortified PDT in cases such as squamous cell carcinoma [39,46], actinic keratosis [37,52,53,55,56,57,58], human psoriasis [36], and follicular mucinosis of the scalp [54]. Research has also been carried out on human glioma cell lines U87 and T98 [44,45], prostate cancer cells [47], and human breast cancer cell lines MCF7 and MDA-MB-231 [43,49]. Furthermore, in experimental research, animal models such as murine models [39,48,49,51], germ-free fetal rat keratinocytes [40], and precancerous lesions in the buccal pouch of hamsters [50] have been used.

The authors of studies assessing the effect of vitamin D and D3 administration (in the form of topical administration) during PDT reported enhanced results compared to un-adjuvant therapy. Response to treatment was variable. Responses depended on the type of case and the patients’ state of health (applies to clinical trials). Most studies focused on dermatological diseases (65%). This may be due to the common use of vitamin D and D3 therapy alone in dermatological cases. It should also be noted that in all clinical trials, the patients were adults over 18 years of age, with people over 50 being qualified more often. Although the therapy itself was a safe procedure, with a reduced number of potential side effects, children and adolescents were not qualified to be included in research groups. In this review, 55% of all studies were experimental (preclinical) in cell lines and animal models. This demonstrates that vitamin D-assisted PDT is a relatively new methodology that is still being studied and remains in the experimental stage. Articles from inception (1979) to 2022 were included in the review. The oldest article is from 2002; i.e., the research spans 20 years.

Our review shows that a much more frequent form of application is topical application of vitamin D and D3 (in 55% of cases, cream or ointment was used). The reason for this may be the number of dermatological cases in which topical application is easier and safer. For example, one clinical case (actinic keratosis) was analyzed in which oral administration was used [37]; in other cases, topical treatment was applied [52,53,55,56,57,58]. Authors who administered oral synthetic vitamin D observed that pre-treatment with high doses improved lesion clearance [37], with few side effects besides a feeling of warmth and a mild tickling sensation during illumination. In the topical application, the response to treatment was high (varying from 6 to even 45% lesion reduction), with good tolerance of tacalcitol. It is worth noting that long-term consumption of a vitamin may itself be toxic to the body and potentially life threatening [71]. Hence, in our opinion, increasing the use of topical application is safer. This information was also taken into account by the authors of [37]; however, they reported that, in fact, many physicians recommend vitamin supplementation in large amounts without risk of toxicity.

This review also included a case of analyzing the effectiveness of a cream compared to an ointment in psoriasis [36]. The authors observed clinical improvement 2 weeks after PDT was present after the application of ointment, which was not observed after the application of cream [36]. Pre-treatment with ointment aided in recovery. The choice of the carrier in which synthetic vitamin D and D3 is delivered is important because the greater the penetration rate of the drug, the higher the increase in the level of PS and the increase in PDT efficiency.

The third case in human clinical trials was follicular mucinosis of the scalp [54]. A single description of a 59-year-old woman treated with tacalcitol was reported. The ointment application itself was started a month before PDT and continued throughout the therapy period without any side effects. The authors observed a progressive regrowth of hair, suggesting increased PDT efficacy. However, in their final conclusion, they suggested PDT therapy in combination with tacalcitol ointment in diseases are refractory to standard forms of treatment, i.e., using it following classical techniques.

The review attempted to analyze the stage of administration of vitamin D or D3 regarding its positive or negative impact. In most studies (95%) included in the review, vitamins D and D3 (and their analogues) were administered before phototherapy. Additionally, in one case, an attempt was made to continue supplementation during therapy [54], and in another case, the process was continued also after therapy [40]. In a study in which supplementation was carried out both before and during PDT, no side effects were observed in the treated woman. At the end of the therapy, there was a gradual reduction of cuticles and new hair growth. The application of tacalcitol was well tolerated by the patient. The study in which the application of calcitriol was used before and after therapy was an in vitro study [40]. The addition of calcitriol to the cell solution after irradiation allowed the observation of the effect of the active form of vitamin D3 on PDT-induced apoptosis. It was observed in the study that treatment of cells with calcitriol after PDT increased the degree of DNA fragmentation compared to untreated cells. The enhanced DNA fragmentation effect was observed in cells that had been injected with calcitriol immediately after irradiation. The supplementation and applications of vitamins D and D3 (and their analogues) were most often implemented before phototherapy; one of the reasons for this phenomenon could be the fact that most of the studies included in the review were human studies. Application or supplementation was carried out locally on the skin; therefore, doctors recommended the application of the ointment/cream in advance (on average 14–15 days). One of the advantages of such a solution is that the patient can apply the preparation to the skin themself, without the supervision of a doctor/nurse. In the case of application after therapy on reddened areas, medical care would be required. It is worth noting that this is a moot point because in the articles, the authors of the research did not provide an explanation of the reason for the time of application. Conducting clinical trials in which the application of vitamin D is implemented after PDT would be able to confirm or contradict our assumptions. Additionally, it would be possible to assess the advantages and disadvantages of each stage of administration in a clear and lucid way.

The review also analyzed the selection of the research group in terms of gender. In the analyzed human studies, both women and men were qualified for research groups in most cases (in 56% of trials). In three cases, male selection was the qualifying criterion. One article was a case report of a woman treated with PDT and vitamin D/D3. The authors did not analyze the effects of vitamin D or D3 supplementation in combination with PDT in relation to the gender of the patients in the evaluation of the results of their studies. In [36], the results of the therapy were similar in both groups when using a cream, ointment, or a different light source. Similar conclusions were presented in [52], where the research group consisted of 10 men and 1 woman. In another study [53], 75% of the research group were men and 25% were women. The description of treatment effects included the characteristics of changes, without taking into account the gender criterion. In the studies presented in [37,55], the authors noted that there were no significant differences in gender in the assessment of treatment results. In studies [56,57,58], male gender was one of the qualifying criteria for the therapy. Based on the above description, it can be concluded that significantly fewer women were qualified for PDT in combination with vitamin D or D3 supplementation compared to the number of men. Perhaps, of the entire group of patients, the majority of patients with dermatological problems are men. Another reason for this phenomenon could be the fact that in all studies, an exclusion criterion was pregnancy. Therefore, a safer variant was the choice of men for therapy than women in whom pregnancy could not be confirmed or excluded, e.g., due to the phase of the menstrual cycle. Nevertheless, the assessment of the gender effects of PDT supplemented with vitamin D or D3 may be a pioneering study that will allow the study protocol to be tailored to each patient (both women and men).

In the clinical trials reviewed, some limitations related to vitamin D and D3 supplementation or application were observed. One of them was the long period of ointment application before PDT. In some cases, this procedure was introduced a month before [54]. Another limitation was the side effects of the therapy, such as facial erythema, scales, and scabs [52]. Additionally, although a given procedure is applicable in a given group of patients, it may not be applicable to all patients.

Animal studies covered 25% of all articles included in the review. The results presented by the authors suggest a positive effect of vitamin D on the effectiveness of PDT. The results presented by the authors of [48] are promising, with the possibility of applying the developed procedures to human research. According to the authors, supplementation with a diet rich in vitamin D may prove to be a much easier method for patients than supplements with a high vitamin content. Researchers in PDT for breast cancer suggest including vitamin D-assisted PDT in human clinical trials as an alternative form of therapy for patients who have not had success with other techniques [49]. The use of the methodologies presented by the authors of [39,50,51] in in vivo studies is an open issue due to the risk of side effects after administration of calcitriol. The method provides hope for patients with non-melanoma skin cancer and squamous cell skin cancer after surgery, radiotherapy, and chemotherapy. We believe that further studies will confirm this in the coming years.

In vitro studies in cell lines covered 30% of articles included in the review [40,43,44,45,46,47]. This type of test is a precursor to in vivo testing. Although the specific test scheme and its effects are satisfactory, it does not determine similar effectiveness in in vivo tests. The cited experimental work indicates a greater effectiveness of PDT after the application of synthetic vitamin D derivatives; this topic must be researched further, perhaps first in animal models, and then in human patients (especially in relation to prostate [47], breast [43], or glioblastoma [44,45] cancers). In in vitro studies (being tested), the dose escalation/reduction process is not as risky as in human clinical studies. The development of an effective in vitro methodology provides the opportunity to increase the effectiveness of the therapy in in vivo clinical studies.

The presented positive effect of vitamin D supplementation in PDT can be assessed in relation to therapy assisted with other vitamins. An example of such therapies is vitamin A [72], E [73], K [74], and C [75] therapy. Vitamins A, E, and C are natural antioxidants which are used as anti-cancer supplements. Similarly to vitamins D and D3 (and their analogues), their influence on the effectiveness of PDT has been studied. In a study by Mahmood et al., the effect of vitamin A in photodynamic therapy of rhabdomyosarcoma cells was evaluated [72]. Vitamin A analogues were applied prior to the photosensitive application and irradiation. The analogues used were retinyl acetate, retinoic acid, and retinyl palmitate, which were applied at various concentrations.

Studies show that pretreatment with vitamin A derivatives reduced the tumor response to PDT. The reason for this phenomenon is the fact that vitamin A has antioxidant properties. PDT, in turn, initiates oxidative stress in cells, leading to the formation of reactive oxygen species. Antioxidants disrupt the action of free radicals, reducing the effectiveness of PDT.

In a study by Mielnikova et al., the effect of the vitamin E analogue on the enhancement of the photodynamic effect was assessed. The studies were carried out on human tumor xenografts [73]. The vitamin E analogue was the water-soluble Trolox when applied with the drug. In the study, a significant delay in tumor growth was observed after the application of Trolox and PDT. The authors made an attempt to explain the cause of this phenomenon. One of the reasons could be that Trolox, which mediated the radical pathway, could also act as a singlet oxygen transmitter, the concentration of which decreases with the progression of PDT. Another explanation may be the fact that Trolox as an antioxidant inhibited the growth of cancer cells after the action of PDT, which initiated an increase in the permeability of blood vessels to Trolox [73].

Another example of assisted therapy is the application of vitamin K3; in [74], rhabdomyosarcoma cells were used. A significant cell destruction effect was observed in this study after vitamin K3 was administered as a neoadjuvant [74]. The combination of a chemotherapeutic drug (CDDP-cisplatin) with vitamin K3 and PDT was also observed to increase the combined efficacy.

In the literature, there are also known cases of vitamin C application before the irradiation process. In [75], the authors used melanoma cells that were incubated at various concentrations one hour before irradiation. It was observed in the experiment that vitamin C supplementation increased cell viability by 10%. The authors showed that vitamin C supplementation caused a significant reduction in cytotoxicity induced by photodynamic treatment in melanoma cells [75]. Pre-treatment with vitamin C reduced the rate of cell death following PDT.

From the review of other forms of PDT enhancement (with vitamins A, K, E, and C), which do not always positively affect the effectiveness of PDT, therapy with vitamins D and D3 (and their analogues) is much more effective.

## 5. Conclusions

In order to improve the quality of PDT research, combination therapies that can improve the PDT method by increasing its effectiveness have been sought for several decades. An example of such a combination therapy is the use of calcitriol, calcipotriol, and tacalcitol on lesions or diseased cells. This review of the studies that has been conducted shows that the combination of vitamin D or D3 with PDT may improve the effectiveness of the method by increasing the accumulation of protoporphyrin via 5-ALA. In addition, PDT supplemented with vitamin D or D3 can enhance cell differentiation, thereby increasing the effectiveness of the treatment. These findings suggest that PDT enhanced with vitamin D and D3 yields a more effective and selective therapeutic response. Due to the fact that all the presented clinical trials used adult patients, the analysis confirming the possibility and effectiveness of using this therapy in young patients remains an open field for doctors and researchers to explore. The proposed therapy model should be further investigated in future clinical trials.

## Figures and Tables

**Table 1:** Description of study characteristics.

Type of Case	Type of Application	Form of Vitamin D	Stage of Administration	Type of Photosensitizer	The Source of Light	Effect on PDT Efficacy (Comments)	Reference
in vitro studies: germ-free fetal rat keratinocytes	application of calcitriol to the cell solution	medium containing calcitriol, final concentration $\left[10^{-6}M\right]$	before and after phototherapy	tetrasulfonate (AlPcTs)	500 W halogen lamp (SX-UI 500 JH; Ushio, Tokyo, Japan)	The addition of calcitriol enhanced DNA fragmentation of cells, thus improving the effectiveness of PDT.	[40]
in vitro studies: human breast cancer cell lines MCF7 and MDA-MB-231	application of calcitriol to the cell solution	calcitriol $[10^{-8}M,\;10^{-10}M,\;$$10^{-12}M,\;10^{-14}M,\;$ $10^{-16}M]$	before therapy	hematoporphyrin derivatives (HPD)	diode laser with a power density of 7.5 J/cm^2^ (XD635AB; Xingda, Guilin, China)	Calcitriol improved the efficacy of PDT by increasing the PpIX levels in cells.	[43]
in vitro studies: human glioma cell lines U87 and T98	application of calcitriol to the cell solution	calcitriol $[10^{-8}M,\;10^{-10}M,\;$$10^{-12}M,\;10^{-14}M]$	before therapy	5-aminolevulinic acid (ALA)	laser with a power density of 30 mW/cm^2^ (XD-635AB; Xingda, Guilin, China)	Calcitriol treatment of glioblastoma cells selectively increased PpIX levels and increased ALA-induced phototoxicity. Additionally, the administered calcitriol significantly increased the number of tumor cells killed after ALA-PDT treatment.	[44,45]
in vitro studies: human squamous cell carcinoma A431 cells	application of calcitriol to the cell solution	calcitriol $\left[10^{-9}-10^{-6}\frac{\mathrm{mol}}{L}\right]$	96 h before therapy	methyl aminolevulinate (MAL)	fluorescent lamps (Model 3026; Applied Photophysics, London, UK) in the wavelength range 370–450 nm	Calcitriol enhanced PDT.	[46]
in vitro studies: LNCaP prostate cancer cells	application of calcitriol to the cell solution	calcitriol and its analogues (R0-25-9022 and R0-26-2198) [10 mM, 1 nM, 10 nM,1 nM]	96 h before therapy	5-aminolevulinic acid (ALA)	argon laser with a wavelength of 514 nm (Coherent, Inc., Santa Clara, CA, USA)	Calcitriol and its analogues significantly increased ALA–PpIX accumulation in cells	[47]
animal studies: human squamous cell carcinoma cell line	diet or systemic administration	one of three forms: • D3: cholecalciferol $\left[40\frac{\mu g}{\mathrm{kg}}\right]$ • monohydroxy D3: calcidiol(25(OH) D3) $\left[250\frac{\mu g}{\mathrm{kg}}\right]$ • dihydroxy D3: calcitriol, (1,25(OH)_2_ D3) $\left[1\frac{\mu g}{\mathrm{kg}}\right]$	diet: 10 days before phototherapy systemic administration: 3 days before	5-aminolevulinic acid (ALA)	633 nm noncoherent light source (LumaCare Products, Newport Beach, CA, USA)	Tumor cells treated with D3 and monohydroxy D3 showed an approximately 2.5- and 3-fold increase in PpIX (protoporphyrin IX) levels compared to vehicle control. Tumors treated with dihydroxy D3 showed an approximately 3.5-fold increase in PpIX (protoporphyrin IX) levels compared to vehicle control. Research showed a clear pattern of increase in cell death induced by ALA-PDT (5-aminolevulinic acid-photodynamic therapy) with vitamin D3 pretreatment.	[48]
animal studies: murine model of breast cancer	intraperitoneally	calcitriol $\left[1\frac{\mu g}{\mathrm{kg}}\right]$	3 days before	5-aminolevulinic acid (ALA)	633 nm noncoherent light source (LumaCare USA, Newport Beach, CA, USA)	Increased cell death was observed in tumors injected with calcitriol prior to ALA-PDT compared to ALA-PDT alone. ALA with calcitriol treatment induced 3.3 ± 0.5-fold increase in intracellular PpIX levels.	[49]
animal studies: squamous cell skin cancers	topically/Intraperitoneally	calcipotriene 0.005% $\mathrm{calcitriol}\;\left[3\frac{\mu g}{g}\right]$ deep tumors: $\mathrm{systemic}\;\mathrm{vitamin}\;D\;\mathrm{in}\;\mathrm{PBS}\;\left[1\frac{\mu g}{\mathrm{kg}}\right]$	3 days before	5-aminolevulinic acid (ALA)	633 nm non-coherent light source (LumaCare Products, Newport Beach, CA, USA)	There was a 10-fold increase in the accumulation of ALA protoporphyrin-IX (PpIX) in neoplastic cells due to changes in the expression of porphyrin synthesis enzymes.	[39]
animal studies: precancerous lesions in the buccal	ointment	calcipotriol 0.005% [100 µL]	every 24 h 3 times	5-aminolevulinic acid (ALA)	LED with 640 nm wavelength	Pre-conditioning of precancerous lesions with calcipotriol affects the amount of PpIX, which may improve the efficacy of PDT.	[50]
animal studies: non-melanoma skin cancer mouse models	topical for the skin	calcitriol $\left[3\frac{\mu g}{g}\right]$	3 days before	5-aminolevulinic acid (ALA)	633 nm noncoherent light source (LumaCare USA, Newport Beach, CA, USA)	Histological examination of tumor tissues from combination therapy (calcitriol + ALA-PDT) showed pyknotic/shrunken testes, reduction of collagen, and growth of dead areas.	[51]
human studies: human psoriasis	cream or ointment	calcipotriol [0.005%]	6 days before	5-aminolevulinic acid (ALA)	635 nm diode laser (HPD 7401, High Power Devices, Inc., North Brunswick, NJ, USA)	In a combination of ALA-PDT therapy with calcipotriol, there was an improvement in the clinical response in psoriatic plaques.	[36]
human studies: actinic keratoses	ointment	calcipotriol $\left[50\frac{\mu g}{g}\right]$	15 days before	methyl aminolevulinate (MAL)	daylight-mediated photodynamic therapy (DL-PDT)	There was a 15% increase in overall response to treatment with DL-PDT in combination with calcipotriol compared to DL-PDT alone.	[52]
human studies: actinic keratoses	ointment	tacalcitol $20\;g\;\left[4\frac{\mu g}{g}\right]$	15 days before	5-aminolevulinic acid (ALA)	630 nm diode (S630, AlphaStrumenti, Milan, Italy)	The combination of PDT with tacalcitol was more effective than the practiced PDT alone. The percentage reduction in the total number of lesions was 44.4%.	[53]
human studies: follicular mucinosis of the scalp	ointment	tacalcitol $\left[4\frac{\mu g}{g}\right]$	1 month before and continued throughout the treatment period	5-aminolevulinic acid (ALA)	ded diode with a wavelength of 630 nm	Applied PDT with tacalcitol effectively reduced inflammation and increased the penetration of 5-ALA into the skin.	[54]
human studies: actinic keratoses	oral	cholecalciferol 10,000 IU	before therapy	5-aminolevulinic acid (ALA)	blue light (10 mW/cm^2^, Blu-U, Sun/DUSA Pharmaceuticals)	Oral vitamin D3 therapy before PDT led to an 18% increase in response to treatment. An increase in the effectiveness of the therapy (by 11%) in removing lesions was also observed.	[37]
human studies: actinic keratosis	cream	calcipotriol 0.005%	2 times a day for 2 weeks before therapy	methyl aminolevulinate (MAL)	red diode lamp (dose 37 J/cm^2^)	Topical therapy with calcipotriol before PDT enhances cell differentiation and apoptosis, thereby increasing the effectiveness of treatment.	[55]
human studies: actinic keratoses	ointment	calcitriol $\left[3\frac{\mathrm{mg}}{g}\right]$	14 days before	methyl aminolevulinate (MAL)	daylight-mediated photodynamic therapy (DL-PDT)	The effectiveness of the therapy with calcitriol was higher by 6.11% compared to the therapy without calcitriol.	[56]
human studies: actinic keratoses	ointment	calcipotriol $\left[50\frac{\mu g}{g}\right]$	15 days before	methyl aminolevulinate (MAL)	-	After 12 months, PDT in combination with calcipotriol was safer and more effective (by approximately 27%) compared to conventional PDT.	[57]
human studies: actinic keratosis	ointment	calcipotriol $\left[50\frac{\mu g}{g}\right]$	15 days before	methyl aminolevulinate (MAL)	red light-emitting diode (LED) (Aktilite; PhotoCure, Oslo, Norway)	The use of PDT with calcipotriol doubled the number of actinic keratoses compared to untreated PDT.	[58]

Note: tacalcitol—synthetic vitamin D3; calcipotriol—synthetic derivative of calcitriol, the active form of vitamin D.

**Table 2:** Summary of studies reviewed.

Type of Study	Number	Type of Case	Number	Method of Delivery of Synthetic Vitamin D or D3 *	Number	Type of Photosensitizer	Number
human studies	9	actinic keratoses	5	ointment/cream	11	5-ALA	11
in vitro cell line	5	squamous cell skin cancers	3	application to the cell solution	5	MAL	6
animal studies	5	breast cancer cell line	2	systemic/intraperitoneally	3	Other	2
		glioma cell lines	1	oral/diet	2		
		psoriasis	1				
		other (individual cases)	7				

*—two studies reported two types of application.

## Data Availability

The data presented in this study is available by request from the corresponding author.
